# Coexistence in Field Samples of Two Variants of the Infectious Salmon Anemia Virus: A Putative Shift to Pathogenicity

**DOI:** 10.1371/journal.pone.0087832

**Published:** 2014-01-30

**Authors:** Constanza Cárdenas, Marisela Carmona, Alicia Gallardo, Alvaro Labra, Sergio H. Marshall

**Affiliations:** 1 Núcleo Biotecnología Curauma, Pontificia Universidad Católica de Valparaíso, Valparaíso, Chile; 2 Laboratorio de Patógenos Acuícolas, Núcleo Biotecnología Curauma, Pontificia Universidad Católica de Valparaíso, Valparaíso, Chile; 3 Laboratorio de Genética e Inmunología Molecular, Instituto de Biología, Facultad de Ciencias, campus Curauma, Pontificia Universidad Católica de Valparaíso, Valparaíso, Chile; 4 Servicio Nacional de Pesca y Acuicultura, Valparaíso, Chile; 5 Fraunhofer Chile Research, Santiago, Chile; University Hospital San Giovanni Battista di Torino, Italy

## Abstract

Genetic reassortment plays an important role in the evolution of several segmented RNA viruses and in the epidemiology of their associated diseases. In particular, orthomyxoviruses show rapid fluctuation in the proportion of viral variants coexisting in an infected individual, especially under strong selective pressure. This is particularly relevant in salmon production carried out under confined and stressful conditions where one of the most feared pathogenic agents is the Infectious Salmon Anemia Virus, an orthomyxovirus family member whose biological behavior is only recently beginning to be understood. Pathogenicity of the virus has been mainly associated with deletions of the HPR region in coding segment 6 and the presence or absence of a specific insertion in a key region in coding segment 5. In this study we report, for the first time in Chile, the coexistence of two variants in fully asymptomatic fish. Of five samples analyzed, two were identified as the non-pathogenic variant, HPR0, and two as the highly pathogenic HPR7b variant, though with no clinical signs detectable in the fish. Interestingly, one of the samples unequivocally carried both variants, again without any clinical signs. Considering that in none of the samples the typical insertion in coding segment 5 was detected, it is our impression that this may represent a shift from the non-pathogenic HPR0 variant towards the highly infective HPR7b variant. If this were the case, the transition may be triggered first by deleting the corresponding sequence of the HPR region of segment 6, followed by the putative insertion in segment 5 to generate a virulent strain.

## Introduction

Intensive aquaculture has generated a new paradigm in the understanding of fish immunity. The required confinement of large numbers of fish has led to the emergence of many diseases that are very rare or absent in their wild counterparts; suggesting a novel dynamic relationship between the pathogen and its host. One particular example is Infectious Salmon Anemia, a viral disease reported for the first time in 1984 in farmed salmon in Norway [1], slowly spreading to all countries with salmon farming operations: Canada [2]–[4], the USA [5], Scotland [6], the Faroe Islands [7], and Chile [8], [9].

**Figure 1:**
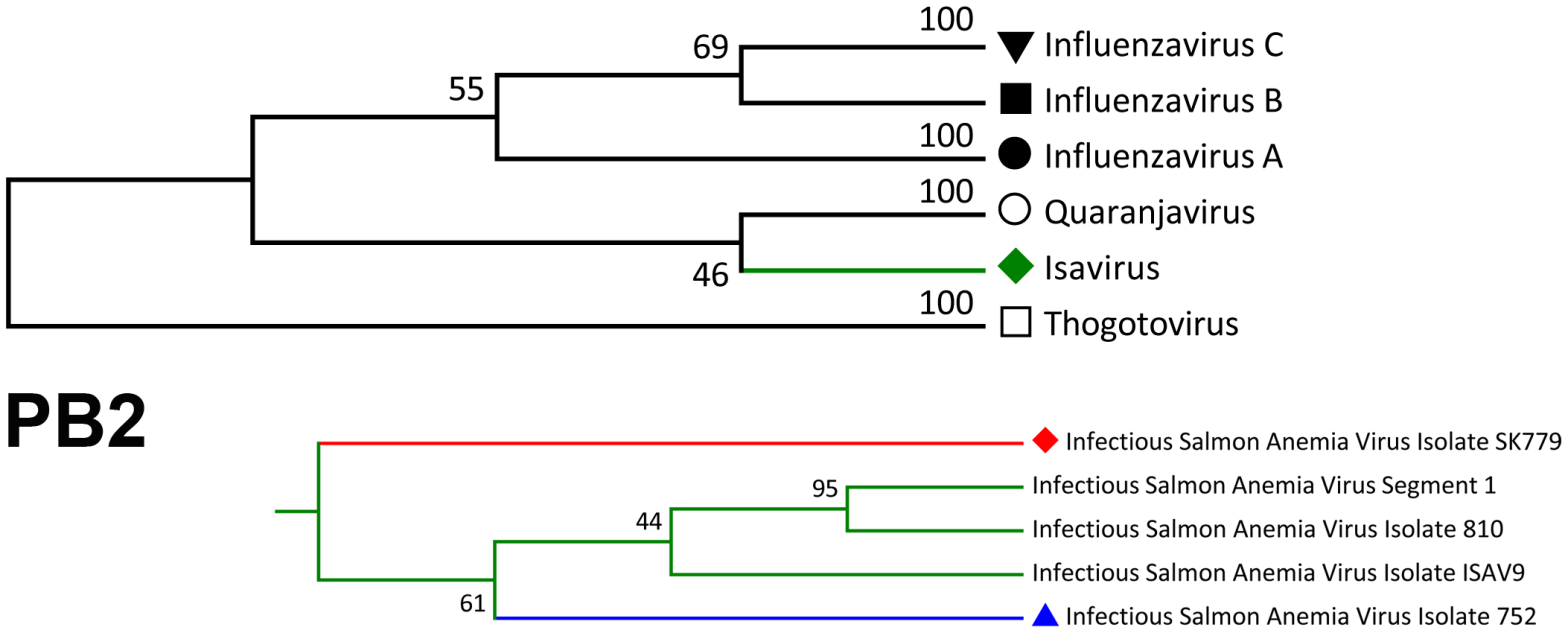
Phylogenetic tree of the *orthomyxoviridae* family (PB2 gene). A sub-tree for the *Isavirus* genus is observed in the lower right corner. The tree was constructed with reference sequences from GenBank database (Accession numbers; Influenza A: NC_004910, HQ117883, AJ40430; Influenza B: NC_002204, AF101982, EF626642; Influenza C: NC_006307, M28061, AF170576; ISAV: NC_006505, AY373381, DQ785183, EU118815, GU830895; Quaranjavirus: GQ499302; Thogotovirus: Y17783). The tree was inferred by using the Maximum Likelihood method based on the Tamura-Nei model with a bootstrap test (1000 replicates) using MEGA 5 software [33].

The etiological agent of Infectious Salmon Anemia is an enveloped RNA virus, the only member of the Isavirus genus belonging to the orthomyxoviridae family (ISAV) [10], [11] (Figure 1). It infects salmonid species almost exclusively, with *Salmo salar* being the main target in Chile.

The ISAV genome is composed of eight single-stranded RNA segments with negative polarity, which codifies for 10 specific proteins. As with the influenza virus, variability and pathogenicity are mostly associated with the two envelope glycoproteins, which for ISAV are hemaglutinin esterase (HE) and a fusion protein (F) encoded in segment 6 and 5, respectively [12]–[18].

A highly polymorphic region, HPR, of the segment 6 coded HE protein has played a pivotal role in classifying the different infection deletion variants reported in the field [12], [14], [19], ranging from the non-pathogenic HPR0 variant containing the complete 35 amino acid region up to the 23 amino acid deletion, represented by HPR7b, the most aggressive variant detected in Chile. The HPR0 variant is believed to be the parental strain [16], [20], [21], [22], since it is not able to produce the disease *in vivo*, although it has not been possible to grow it *in vitro*.

For the segment 5 coded F protein, two events seem to be important in defining its pathogenic potential: a putative insertion near the cleavage site of the protein and a key amino acid substitution (Q_266_/L) [19]. Pathogenic variants carry either one of four different insertions (IN1-IN4) or the substitution of the amino acid Q_266_ for L. The non-pathogenic variant HPR0 is associated with the absence of insertion and Q_266_.

Normally, one single variant is detected either in an epizootic outbreak or in fishery disease surveillance, based upon the standard qRT-PCR procedure described by Snow et al [23]. This test is based upon the amplification of genomic viral segment 8, that includes a high conserved region and is the standard test accepted by the World Organization for Animal Health (OIE). Under this condition the coexistence of two variants has not been reported in natural infections.

We have developed two highly specific techniques [24], [25] to further characterize novel variants in the field, which may allow us to determine more than one variant in a single sample.

The present report comprises the analysis of field samples from asymptomatic fish in southern Chile as part of the Specific Health Surveillance and Control of Infectious Salmon Anemia Virus (PSEVC-ISA) a program developed by Sernapesca (National Fisheries and Aquaculture Service). We demonstrate the coexistence of two different variants in a single organ pool: the non-pathogenic HPR0 and the highly pathogenic HPR7b.

Although the pathogenic variant should have had the expected insertion in segment 5, this was lacking in all cases analyzed, which is suggestive of an intermediate stage in pathogenicity development.

## Results

### Standard Determination of the Presence of the ISA Virus

Following standard diagnostic procedures, the five target samples were analyzed for genomic segment 8 [23], as shown in Table 1. All samples were positive for ISAV.

**Table 1:** qRT-PCR analysis for genomic segment 8 based on Snow et al. [23].

Sample	Ct Segment 8 Snow	Diagnostic
GIM-1	26.01	Positive
GIM-2	26.01	Positive
GIM-3	33.62	Positive
GIM-4	33.18	Positive
GIM-5	32.23	Positive

### Further Characterization of Genomic Segments 6 and 5

Considering that the samples tested were positive for segment 8, genomic segments 6 and 5 were characterized using PCR amplification and sequencing, as shown in Table 2. The sequence data for segment 6 of sample GIM-3 presented reading problems, with confusing signals in the polymorphic region possibly due to a poor amplification for a low viral concentration, which usually happens with variant HPR0.

**Table 2:** Summary of sequencing results for genomic segments 6 and 5.

Sample	Segment 6 HPR variant	Segment 5‡	
		Insert	AA_266_
GIM-1	**0**	No Insert	Q
GIM-2	**0**	No Insert	Q
GIM-3	**ND**	No Insert	Q
GIM-4	**7b**	No insert	Q
GIM-5	**7b**	No Insert	Q

As reference sequences we used EU118820 for HPR0 variant and GU830900 for HPR7b variant from the GenBank database.

‡ Referred to the presence of insert-4 (IN4) and the specific amino acid (AA) in position 266.

ND: not determined.

Due to the fact that initial sequencing analysis after PCR amplification showed that at least two of the samples unquestionably contained variant HPR7b for segment 6 but lacked the presence of the insertion IN-4 in segment 5, samples were submitted to both High Resolution Melting (HRM) and Denaturing Gradient Gel Electrophoresis (DGGE) for further characterization.

### High Resolution Melting Analysis

**Figure 2:**
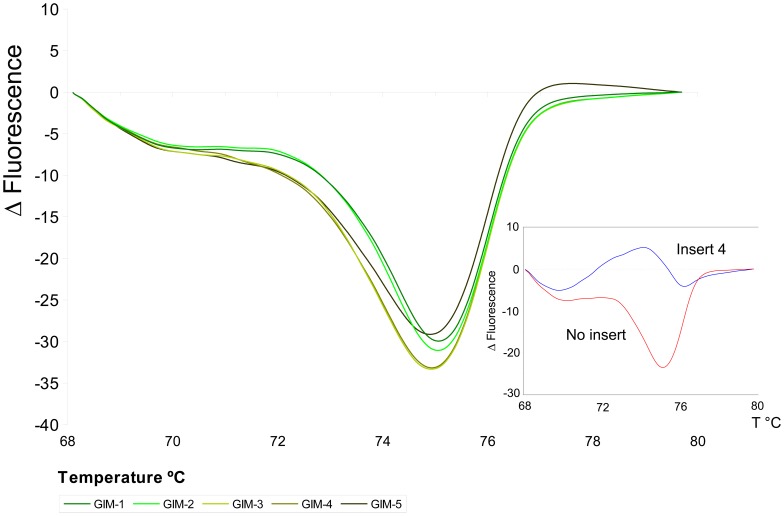
High Resolution Melting Profiles for segment 5. The lower right corner shows the standard reference variants, with No Insert: red line, and with Insert 4: blue line. The analyzed samples all matched with the No Insert variant.

The HRM melting temperature results were analyzed and the profile curves were generated for the field samples and compared with the reference standards for the Chilean epizootic variants [24]. The melting temperatures and HRM profiles for segment 5 (Figure 2) were consistent with the sequencing analysis. All samples were confirmed to belong to the HPR0 variant.

**Figure 3:**
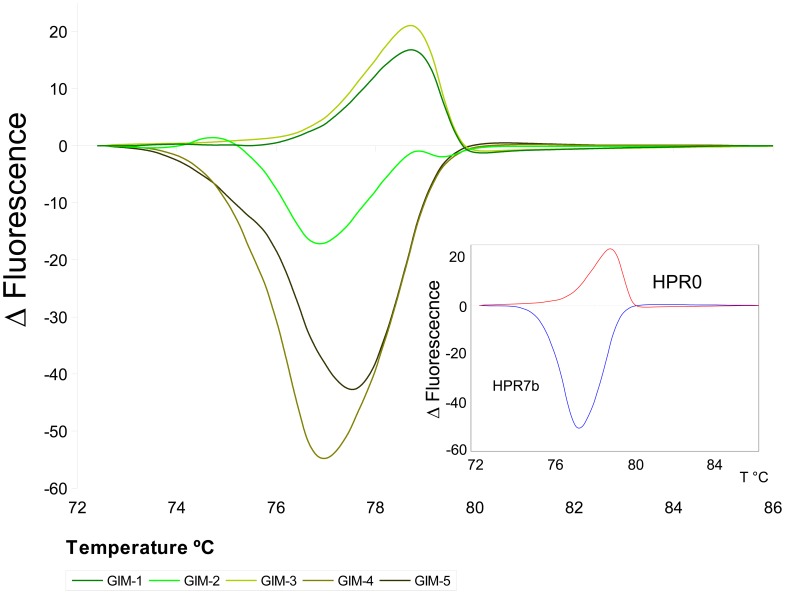
High Resolution Melting Profiles for segment 6. The lower right corner shows the standard reference variants; HPR0: red line, and HPR7b: blue line. Two samples, GIM-1 and GIM-3 matched the HPR0 variant profile; another two, GIM-4 and GIM-5 matched the HPR7b variant profile; GIM-2 sample does not match any of the two profiles. This behavior is coherent with sequencing results, in which GIM-2 showed an overlapping sequence of the two variants (HPR0 and HPR7b).

For segment 6 the profiles matched the reference profiles for HPR0 and HPR7b with the exception of sample GIM-2 which displayed a distinctive profile (Figure 3). Table 3 shows the HRM results.

**Table 3:** Summary of HRM results for field samples.

Reference standards Melting Temperature				Sample	HRM variant Segment 5		HRM variant Segment 6	
Seg 5		Seg 6			MT*	Insert	MT*	HPR
**MT** *	**Insert**	**MT** *	**HPR**	GIM-1	74.1	No	80.0	HPR0
74.0	No	79.2	HPR0	GIM-2	74.1	No	**	**
				GIM-3	73.9	No	79.1	HPR0
76.0	Insert 4	76.5	HPR7b	GIM-4	74.0	No	76.3	HPR7b
				GIM-5	73.9	No	76.5	HPR7b

* Melting Temperature in °C.

** There are two values for Melting Temperature and the profile does not match the reference variants (Figure 2).

### Denaturing Gradient Gel Electrophoresis Analysis

For DGGE analysis multiple bands were obtained for each sample [25] and were all sequenced (Table 4).

**Table 4:** Sequencing results DGGE bands.

Samples	Segment 5	Segment 6	
	DGGE band	DGGE band	ISA variant
GIM-1	1.1	1.1	0
	1.2	1.2	0
GIM-2	2.1	2.1	0
	2.2	2.2	0
		2.3	0/7b**
GIM-3	3.1	3.1	0
	3.2	3.2	0
GIM-4	4.1	4.1	7b
	4.2	4.2	7b
		4.3	7b
GIM-5	5.1	5.1	7b
	5.2	5.2	7b
		5.3	7b

** Overlapping of HPR0 and HPR7b variant sequence.

**Figure 4:**
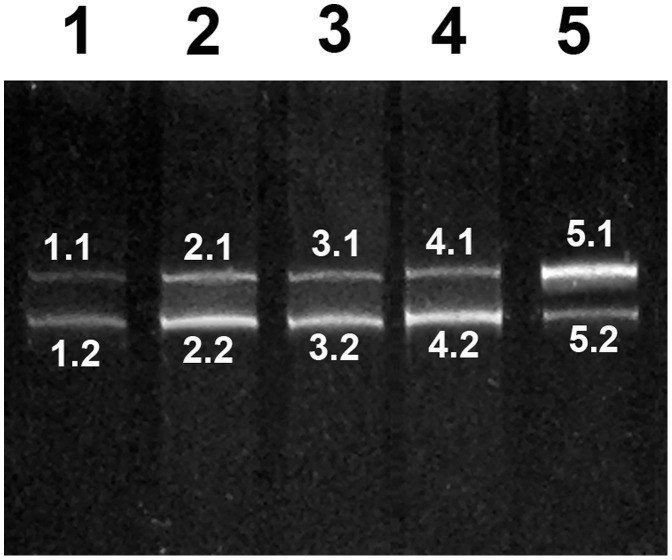
DGGE band profile for segment 5. Lanes 1–5 corresponds to samples GIM-1 to GIM-5. The sequencing results for each band are summarized in Table 4.

For segment 5, the same band profile was obtained for all samples (Figure 4). Although two bands were obtained in all cases, the sequencing showed that they were identical and unequivocally corresponded to a genomic segment 5 without an insertion and the coding for a glutamine residue at position 266 (Q_266_).

**Figure 5:**
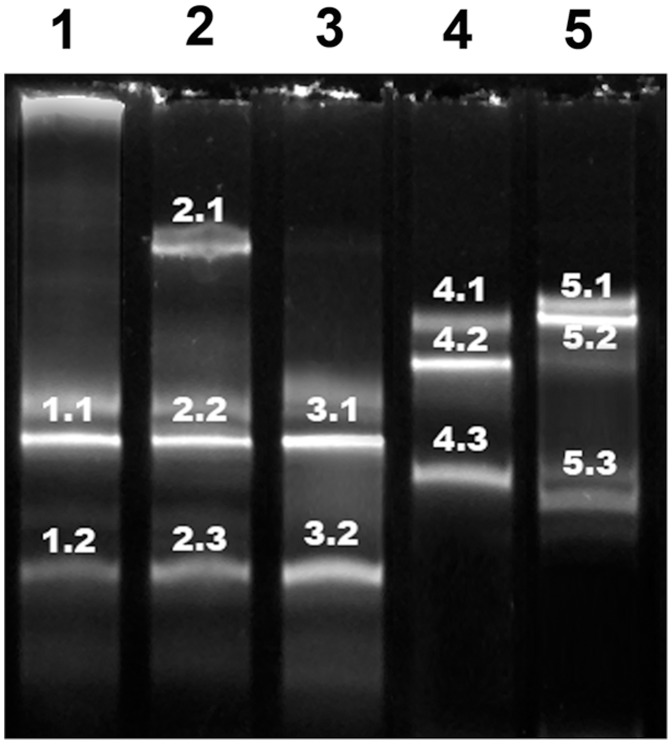
DGGE band profile for segment 6. Lanes 1–5 corresponds to samples GIM-1 to GIM-5. The sequencing results for each band are summarized in Table 4.

For segment 6, though a heterogeneous band profile was obtained for each sample (Figure 5), individual isolation and sequencing confirmed that they corresponded to the two variants detected in the initial sequencing analysis (Table 4). Surprisingly, the sequencing of one DGGE band from one of the samples (GIM-2 Table 4) revealed an overlap of the two variant sequences (Figure S1).

## Discussion

Diagnostic procedures to detect pathogenic agents in the aquaculture world mainly focus on defining a positive or a negative case primarily based on alternative PCR procedures. Intrinsically, the procedure may limit the interpretation of a positive result if it corresponds to a single strain or variant of the agent. Nevertheless, in the case of the ISAV, as expected for a member of the orthomyxovirus family, genetic variability should be an important issue for consideration. Indeed, among the multiple variants detected in Chile, the non-pathogenic HPR0 is the most predominant form, and the same is seen in reports from other countries. As hypothesized, HPR0 may be considered the benign wild-type strain from which virulent forms are derived [20], [22]. Therefore, the possibility exists that evolving viral populations in fish under confined and stressing conditions shift towards pathogenicity and more than one variant be detected simultaneously in the general population.

In this report, two ISAV variants have been detected for the first time in a pooled sample of asymptomatic fish. Intriguingly, one of the variants is the most aggressive form of the virus detected in Chile (HPR7b), responsible for full development of the disease *in vivo*, though in this case it has been identified in fully asymptomatic specimens. Since pathogenicity in Chilean field samples has always been associated with a deletion in the HPR region of segment 6 with a concomitant insertional event in segment 5 [26], [27], it is then tempting to think that progression to pathogenicity might very well be a two-stage process, involving first a deletion in the HPR region which in turn might constitute, in time, a triggering induction of a transpositional event from segment 2 to segment 5 as the IN4 insertion associated with the virulent phenotype HPR7b. Therefore, samples reported here might be in the transitional stage and as such; do not express signology associated with the disease. Another alternative possibility is that up to now, although ISAV has been detected in fresh water specimens, both in natural as well as in confined environments [8], [28], [29] the disease has never been reported. Thus, the possibility exists that fresh water conditions restrain full viral expression and the concept of latency or persistence might play a key role under these conditions. Our laboratory is defining strategies to approach these two possibilities.

Since general ISAV diagnosis is based on the detection of highly conserved sequence of viral segment 8, we decided to further analyze potential variability of the two genomic segments directly associated with viral pathogenicity, segment 6 and segment 5 by using two very sensitive and highly complementary techniques, standardized in our lab, HRM and DGGE.

Sequencing for segment 5 from the five samples analyzed yielded identical results for all samples, without the insertion associated with pathogenicity and the presence of glutamine as a key amino acid in position 266, two distinctive features of the HPR0 variant. These observations were confirmed by HRM analysis (Figure 2).

In the case of segment 6, unexpected results were observed. Two out of the five samples correspond to the virulent HPR7b variant despite the features of segment 5 described above. Of the three remaining samples, two correspond solely to the HPR0 variant, while the third presented the two variant sequences simultaneously (GIM-2 in Table 2). HRM, DGGE and re-sequencing further validated these observations (Figure 3).

The stressful conditions of the net/pen rearing of salmonid fish constitute an ideal environment for pathogen evolution. The results reported here are consistent with this possibility. In the ISAV world, it is an accepted fact that a virulent variant carries a deletion in the HPR region of genome segment 6 and at the same time it may either contain an insertion and/or a key amino acid change in genome segment 5.

There are two possible molecular events associated with the acquisition of virulence. Deletions primarily associated with the HPR region of segment 6 constitute a mutation issue and/or an insertion. It is a more complex process associated with reassortment, recombination and even transposition. Thus, deletions involving segment 6 in particular may be the triggering event of evolution towards a virulent genotype.

As such, HPR0 may very well be the template from which the transformation from non-pathogenicity to pathogenicity initiates. Selective pressure of different origins might contribute to this process. From many alternatives, our results agree with this interpretation, pointing to the fact that the aforementioned shift does occur by an initial triggering of a deletion in the HPR region of segment 6 followed at least by a site-specific insertion in segment 5. These two consecutive modifications could constitute the minimal virulent profile associated with disease development.

## Materials and Methods

**Figure 6:**
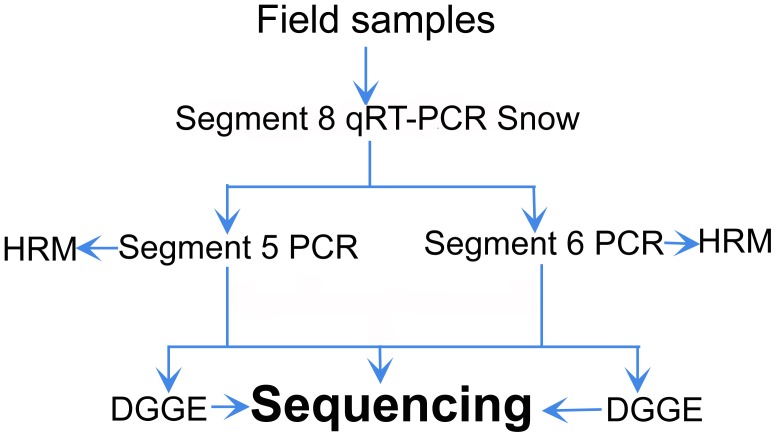
Diagram of the PCR-based experimental methodology.

A diagram of the experimental methodology used is shown in Figure 6.

### Samples

As part of the Specific Health Surveillance and Control of the Infectious Salmon Anemia Virus (PSEVC-ISA) field samples were collected from 16 different centers in the XI region of the country. Five of them were positive for the ISAV-HPR0 variant. Sampling was carried out by Sernapesca following the guidelines given in the Procedure for Sampling Aquatic Animals (LABD/NT1 [30]) which is based on the guidelines of the World Organization for Animal Health (OIE). After necropsy analysis, samples were taken from gills, heart and kidney of apparently healthy fish. Tissues were collected as pools of three fish from the same culture unit and maintained in RNAlater (Ambion) until processing.

### Ethics Statement

The samples were collected by official veterinarians in accordance with the specific standards defined by the officially responsible entity in Chile (Sernapesca). Samples were provided by Sernapesca and processed at the *Laboratorio de Patógenos Acuícolas* which is a reference lab for ISAV diagnosis in accordance with resolution 1448 of 6^th^ July of 2011 issued by Sernapesca. It therefore complies with the standards and procedures established by OIE.

### RNA Extraction and cDNA Synthesis

Total RNA extraction starts with tissue homogenization by using the Magnalyser (Roche) followed by the extraction with the RNeasy MiniKit (Qiagen, MD, USA). cDNA synthesis was performed with random hexamers of primers (Fermentas, USA) as per standard protocols recommended by the supplier.

### Determination of the Presence of ISAV

ISAV presence was confirmed in our laboratory by using the qRT-PCR for segment 8 in accordance with Snow, 2006, applying a normal diagnoses process [23] (Table S1).

### PCR Primary Amplification

**Figure 7:**
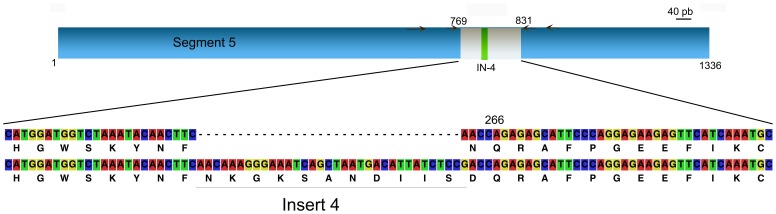
Target for PCR amplification on segment 5 as template. Insert 4 and amino acid in position 266 are indicated. Arrows indicate primer locations.

**Figure 8:**
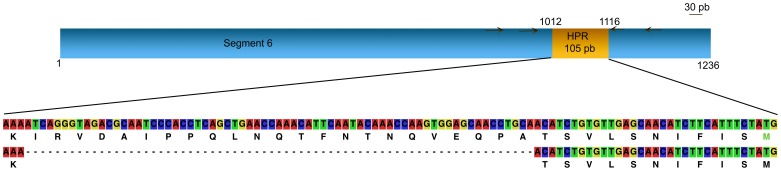
Target for PCR amplification on segment 6 as template. HPR region is indicated. Arrows indicate primer locations.

After confirmation of the presence of ISAV, different samples were amplified for segment 5 and segment 6 with a first set of primers for each segment (Table S1 and Figures 7 and 8). All products were separated by neutral agarose electrophoresis gel; the bands were excised, purified and sequenced (Macrogen, Seoul, Korea).

### PCR Secondary Amplification

Samples were submitted to a second round of amplification with a specific set of primers (Table S1 and Figures 7 and 8) in order to perform the HRM and DGGE analyses.

### High Resolution Melting Analysis

Samples were analyzed by HRM in accordance with standardized procedure [24]. Briefly, RNA was extracted with the RNeasy MiniKit (Qiagen, MD, USA). cDNA was synthesized by using hexamers (following standard protocol). Before the HRM reaction, a first PCR amplification was carried out to normalize the conditions. The resultant products were analyzed in a 2% agarose gel, and the bands excised and purified using a gel extraction kit (E.Z.N.A, Omega Bio-tek). The HRM PCR reaction was conducted with a diluted solution of this product (1:1000) and by using the MeltDoctor Kit (Applied Biosystem) with appropriate primers (Table S1). Reference samples were used to analyze the melting curves.

### Denaturing Gradient Gel Electrophoresis Analysis

Samples were analyzed by DGGE in accordance with a previously described procedure [25]. Briefly, 40 uL of GC clamped-amplicons were resolved in 8% acrylamide parallel gels (37.5:1, acrylamide:Bis-acrylamide) using a narrow range of denaturant (30–60%). Electrophoresis was run at a constant 130 V for 90 min and at 56°C using the Bio-Rad D-Code™ Universal Mutation Detection System. The DGGE bands were excised from the original gel and incubated in 100 ul of sterile distilled water at 4°C overnight. A 10 ul aliquot of the elution was used for PCR amplification of the DNA fragments. The PCR products were visualized and the bands were excised and purified with a DNA gel extraction kit (E.Z.N.A, Omega Bio-tek) and sent for sequencing (Macrogen, Korea).

### Bioinformatics Analysis

The sequence data were analyzed using the CLC workbench (CLCbio). The BLAST algorithm [31] was used to find homologous sequences in the GenBank database [32].

## Supporting Information

Figure S1 **Trace data for the overlapping sequences.** Upper panel: comparison of reference sequences (EU11820 and GU830900) with the field samples GIM-1, GIM-2 and GIM-4. Bottom panel: detail of the overlapping moiety.
(TIF)

Table S1 **Primers used for the different assays for each segment.** The primers are the same as those reported in our previous work [21], [22] but the nomenclature has been changed.
(DOCX)
