# Supplementary material for: Coexistence in Field Samples of Two Variants of the Infectious Salmon Anemia Virus: A Putative Shift to Pathogenicity
Source: PLoS One. 2014 Jan 30;9(1):e87832. doi: 10.1371/journal.pone.0087832 (PMC3907575; doi:10.1371/journal.pone.0087832)
Supplement: Table S1 — Primers used for the different assays for each segment. The primers are the same as those reported in our previous work [21], [22] but the nomenclature has been changed. (DOCX) [file pone.0087832.s002.docx]

**Table S1.**

| **Segment** | **Assay** | **Primer** | **Sequence 5’-3’** |
| --- | --- | --- | --- |
| **8** | Diagnostic | ISAS8-02F | **TGCTACACAGCAGGATGCAG** |
|  |  | ISAS8-02R | **CATCTTCTCTGTCGAGCAGGA** |
|  |  | ISAS8-01P* | **FAM-CATCGTCGCTGCAGTTC-MGB** |
| **6** | Primary PCR | ISAS6-02F | **GCCCAGACATTGACTGGAGTAG** |
|  |  | ISAS6-04R | **CTCTAGACTTGTACATGAATGCTG** |
|  | HRM | ISAS6-05F | **TCATGAGGGAGGTAGCATTG** |
|  |  | ISAS6-05R** | **CAATCCCAAAACCTGCTACAC** |
|  | DGGE | ISAS6-06F | **TGAGGGAGGTAGCATTGCAT** |
| **5** | Primary PCR | ISAS5-02F | **TACAACGGAAAGGATTAAGACTG** |
|  |  | ISAS5-02R | **TCTCCTTCTAGCAGCAGGTTC** |
|  | HRM | ISAS5-04F | **ATGGATGGTCTAAATACAACTTC** |
|  |  | ISAS5-03R** | **ACAGCATTTGATGAACTCTTCTC** |
|  | DGGE | ISAS6-06F | **TACCAGACAGGCTAGGGTTC** |

* Probe. ** Reverse primer is the same for HRM and DGGE.
